# Supplementary material for: Leaders in the global banking network: Analysis of the Bank for International Settlements network data
Source: PLoS One. 2025 Oct 31;20(10):e0335506. doi: 10.1371/journal.pone.0335506 (PMC12578327; doi:10.1371/journal.pone.0335506)
Supplement: Attachment — Submitted filename: Response to Reviewers Comments.pdf [file pone.0335506.s001.pdf]

Response to Reviewers Comments.

We respond point-by-point to the reviewer comments. The only substantive issue was changing the abstract of the paper (see #6), which was addressed. Responses are in **bold**.

-Anthony Bonato

Reviewers' comments:

Reviewer's Responses to Questions

Comments to the Author

1. If the authors have adequately addressed your comments raised in a previous round of review and you feel that this manuscript is now acceptable for publication, you may indicate that here to bypass the “Comments to the Author” section, enter your conflict of interest statement in the “Confidential to Editor” section, and submit your "Accept" recommendation.

Reviewer #1: All comments have been addressed

Reviewer #2: All comments have been addressed

No action taken.

2. Is the manuscript technically sound, and do the data support the conclusions?

The manuscript must describe a technically sound piece of scientific research with data that supports the conclusions. Experiments must have been conducted rigorously, with appropriate controls, replication, and sample sizes. The conclusions must be drawn appropriately based on the data presented.

Reviewer #1: Yes

Reviewer #2: Yes

No action taken.

3. Has the statistical analysis been performed appropriately and rigorously?

Reviewer #1: N/A

Reviewer #2: Yes

No action taken.

4. Have the authors made all data underlying the findings in their manuscript fully available?

The PLOS Data policy requires authors to make all data underlying the findings described in their manuscript fully available without restriction, with rare exception (please refer to the Data Availability Statement in the manuscript PDF file). The data should be provided as part of the manuscript or its supporting information, or deposited to a public repository. For example, in addition to summary statistics, the data points behind means, medians and variance measures should be available. If there are restrictions on publicly sharing data—e.g. participant privacy or use of data from a third party—those must be specified.

Reviewer #1: Yes

Reviewer #2: Yes

No action taken.

5. Is the manuscript presented in an intelligible fashion and written in standard English?

PLOS ONE does not copyedit accepted manuscripts, so the language in submitted articles must be clear, correct, and unambiguous. Any typographical or grammatical errors should be corrected at revision, so please note any specific errors here.

Reviewer #1: Yes

Reviewer #2: Yes

No action taken.

6. Review Comments to the Author

Please use the space provided to explain your answers to the questions above. You may also include additional comments for the author, including concerns about dual publication, research ethics, or publication ethics. (Please upload your review as an attachment if it exceeds 20,000 characters)

Reviewer #1: The revised manuscript, now entitled "Leaders in the global banking network: analysis of the Bank for International Settlements network data", has addressed all the concerns that I had with its initial version, and seems almost ready for publication.

The only change that I'd recommend for the manuscript would be to rewrite the abstract to be a more descriptive overview of the study performed and the results obtained. Currently, it's too generic and non descriptive enough to be a concise summary of the work contained in the article, and in my view the abstract should be the best opportunity to convince your future readers of the relevance of your research and to read your work.

Try to start it by adding a quick context of the field, with one sentence as the general context and another one tackling the gap in literature that you plan to address. Next, very briefly introduce your methodology, similar to 'In this work, we address these challenges by proposing a methodology that (...)', although this section of the current abstract is clear enough. Also, finish it by highlighting the paper's most relevant findings explicitly.

A new abstract was included following the reviewer's request. Note that no other further edits were made to the revision.

Reviewer #2: As the authors have addressed the points I raised in my report, I think the article can now be published in PLOS ONE.

No action taken.

7. PLOS authors have the option to publish the peer review history of their article (what does this mean?). If published, this will include your full peer review and any attached files.

If you choose “no”, your identity will remain anonymous but your review may still be made public.

Do you want your identity to be public for this peer review? For information about this choice, including consent withdrawal, please see our Privacy Policy.

Reviewer #1: No

Reviewer #2: No

No action taken
