# Supplementary material for: Leaders in the global banking network: Analysis of the Bank for International Settlements network data
Source: PLoS One. 2025 Oct 31;20(10):e0335506. doi: 10.1371/journal.pone.0335506 (PMC12578327; doi:10.1371/journal.pone.0335506)
Supplement: Attachment — Submitted filename: Response_to_Reviewers_Comments_auresp_3.pdf [file pone.0335506.s002.pdf]

Response to Reviewers Comments.

Additional Editor Comments:

In the previous decision you were asked by the Editorial Office to address the below comment: We note that the use of artificial intelligence (AI) tools and technologies were used in the preparation of this manuscript. As per our policy (https://journals.plos.org/plosone/s/ethical-publishing-practice#loc-artificial-intelligence-tools-and-technologies) these contributions must be clearly reported in a dedicated section of the Methods, or in the Acknowledgements section for article types lacking a Methods section. Therefore, in your revised manuscript please include a declaration that states the following information: (1) the name(s) of any tools used, (2) a description of how the authors used the tool(s), (3) how authors evaluated the validity of the tool's outputs; and (4) a clear statement of which aspects of the study, article contents, data, or supporting files were affected/generated by AI tool usage. Noncompliance with any aspect of this policy will be considered misrepresentation of methods, contributions, and/or results and may result in the rejection of your submission.

Response: In preparing the revised version of our manuscript, we removed all passages that had previously been drafted with the assistance of large language models. The present version (and the previously submitted revision) contains no text generated or assisted by artificial intelligence tools. Accordingly, there are no AI contributions that require disclosure under PLOS ONE's policy.

Reviewer's Responses to Questions

Comments to the Author

1. If the authors have adequately addressed your comments raised in a previous round of review and you feel that this manuscript is now acceptable for publication, you may indicate that here to bypass the "Comments to the Author" section, enter your conflict of interest statement in the "Confidential to Editor" section, and submit your "Accept" recommendation.

Reviewer #1: All comments have been addressed

Response: No changes made to the manuscript.

---

2. Is the manuscript technically sound, and do the data support the conclusions?

The manuscript must describe a technically sound piece of scientific research with data that supports the conclusions. Experiments must have been conducted rigorously, with appropriate controls, replication, and sample sizes. The conclusions must be drawn appropriately based on the data presented.

Reviewer #1: Yes

Response: No changes made to the manuscript.

---

3. Has the statistical analysis been performed appropriately and rigorously?

Reviewer #1: N/A

Response: No changes made to the manuscript.

---

4. Have the authors made all data underlying the findings in their manuscript fully available?

The PLOS Data policy requires authors to make all data underlying the findings described in their manuscript fully available without restriction, with rare exception (please refer to the Data Availability Statement in the manuscript PDF file). The data should be provided as part of the manuscript or its supporting information, or deposited to a public repository. For example, in addition to summary statistics, the data points behind means, medians and variance measures should be available. If there are restrictions on publicly sharing data—e.g. participant privacy or use of data from a third party—those must be specified.

Reviewer #1: Yes

Response: No changes made to the manuscript.

---

5. Is the manuscript presented in an intelligible fashion and written in standard English?

PLOS ONE does not copyedit accepted manuscripts, so the language in submitted articles must be clear, correct, and unambiguous. Any typographical or grammatical errors should be corrected at revision, so please note any specific errors here.

Reviewer #1: Yes

Response: No changes made to the manuscript.

---

6. Review Comments to the Author

Please use the space provided to explain your answers to the questions above. You may also include additional comments for the author, including concerns about dual publication, research ethics, or publication ethics. (Please upload your review as an attachment if it exceeds 20,000 characters)

Reviewer #1: This revision addressed my previous comments concerning the abstract, which is now much clearer and representative of the study. *I believe that the manuscript is sound enough to be published at PLOS One.*

Response: No changes made to the manuscript.
